# Discovery of a Novel Prolactin in Non-Mammalian Vertebrates: Evolutionary Perspectives and Its Involvement in Teleost Retina Development

**DOI:** 10.1371/journal.pone.0006163

**Published:** 2009-07-08

**Authors:** Xigui Huang, Michelle N. Y. Hui, Yun Liu, Don S. H. Yuen, Yong Zhang, Wood Yee Chan, Hao Ran Lin, Shuk Han Cheng, Christopher H. K. Cheng

**Affiliations:** 1 School of Biomedical Sciences, The Chinese University of Hong Kong, Sha Tin, New Territories, Hong Kong, China; 2 Department of Biology and Chemistry, City University of Hong Kong, Kowloon, Hong Kong, China; 3 State Key Laboratory of Biocontrol, Institute of Aquatic Economic Animals, and the Guangdong Province Key Laboratory for Aquatic Economic Animals, Sun Yat-Sen University, Guangzhou, China; Ecole Normale Supérieure de Lyon, France

## Abstract

**Background:**

The three pituitary hormones, viz. prolactin (PRL), growth hormone (GH) and somatolactin (SL), together with the mammalian placental lactogen (PL), constitute a gene family of hormones with similar gene structure and encoded protein sequences. These hormones are believed to have evolved from a common ancestral gene through several rounds of gene duplication and subsequent divergence.

**Principal Findings:**

In this study, we have identified a new *PRL*-like gene in non-mammalian vertebrates through bioinformatics and molecular cloning means. Phylogenetic analyses showed that this novel protein is homologous to the previously identified PRL. A receptor transactivation assay further showed that this novel protein could bind to PRL receptor to trigger the downstream post-receptor event, indicating that it is biologically active. In view of its close phylogenetic relationship with PRL and also its ability to activate PRL receptor, we name it as PRL2 and the previously identified PRL as PRL1. All the newly discovered PRL2 sequences possess three conserved disulfide linkages with the exception of the shark PRL2 which has only two. In sharp contrast to the classical PRL1 which is predominantly expressed in the pituitary, PRL2 was found to be mainly expressed in the eye and brain of the zebrafish but not in the pituitary. A largely reduced inner nuclear layer of the retina was observed after morpholino knockdown of zebrafish *PRL2*, indicating its role on retina development in teleost.

**Significance:**

The discovery of this novel PRL has revitalized our understanding on the evolution of the *GH/PRL/SL/PL* gene family. Its unique expression and functions in the zebrafish eye also provide a new avenue of research on the neuroendocrine control of retina development in vertebrates.

## Introduction

The vertebrate growth hormone (GH) family consists of three pituitary hormones, i.e. GH, prolactin (PRL) and somatolactin (SL), and the mammalian placental lactogen (PL). Because of their similarity in gene structure and protein sequences, they are believed to have evolved from a common ancestral gene through several rounds of gene duplication and subsequent divergence [1]–[3].

Living vertebrates are divided into two lineages: the jawless vertebrates (Cyclostomes) and the jawed vertebrates (Gnathostomes). The jawless vertebrates, represented only by hagfish and lamprey today, are regarded as the basal group of vertebrates. The jawed vertebrates are divided into cartilaginous fishes (Chondrichthyes), bony fishes (Osteichthyes) and tetrapods. The cartilaginous fish are regarded as the basal group of jawed vertebrates [4]. Till now, PRL sequences have been characterized in many different vertebrate species, ranging from the most primitive ray-finned fish to mammals [3]. In contrast to GHs and SLs, PRL sequences exhibit great divergence among different vertebrate taxa and its evolutionary history has long been an intriguing question. Sequence analysis showed that all the tetrapod PRLs possess three conserved disulfide linkages, while the teleost PRLs only contain two disulfide linkages. On the basis of this difference, a hypothesis was thus proposed suggesting that the evolution of PRL can be branched into two lineages, i.e. the tetrapod lineage and the teleost lineage [5]–[7]. This hypothesis can accommodate the PRLs identified in teleosts and tetrapods so far. However several questions regarding PRL evolution and classification remained unanswered. First, when all fish lineages (Chondricthyes, Chondrostei, Ginglymodi and Teleostei) are included in the analysis, the evolutionary picture becomes obscure because the sturgeon (Chondrostei) PRL has three disulfide linkages which is the same as tetrapod PRLs. However, sturgeon PRL shares a higher homology with the teleost PRLs than that of the tetrapod [8]. Second, the occurrence and the complete evolutionary picture of PRLs in vertebrates has not been fully elucidated because of the absence of PRL information in Chondricthyes and Cyclostomes so far. Third, after a single GH with two conserved disulfide linkages was identified in a jawless fish, lamprey (*Petromyzon marinus*), GH was regarded as the ancestor of the GH/PRL/SL family and PRL present in gnathostomes was regarded as the gene duplication and subsequent diversification product of this ancestral GH [9]. However, all GHs contain two disulfide linkages only while the sturgeon and tetrapod PRLs contain three. Thus the question of when PRLs have acquired the additional disulfide linkage and what evolutionary processes they have experienced after the duplication event remains unclear. Considering that Chondrichthyes and Cyclostomes occupy strategic positions in the evolution of bony fish and jawed animals, and that their genome information is now available, these valuable resources render us the possibility of understanding more about the evolutionary history of PRLs in vertebrates.

To date, the genomic sequences of sea lamprey and elephant shark (*Callorhinchus milii*, a cartilaginous fish) have become available. Such sequence information can provide important cues on the evolution of some specific genes. In this study, we have searched the genomes of these two species together with other vertebrate and invertebrate genomes. We have found a new *PRL*-like gene in almost all the non-mammalian vertebrate classes, ranging from cartilaginous fish to tetrapods. Subsequent analysis demonstrates that this novel gene contains five exons and four introns, the same as other members of the *GH* gene family. Phylogenetic analyses show that the protein encoded by this novel gene is more homologous to the previously identified PRLs than to GHs and SLs. Receptor transactivation assay demonstrates that this protein is biologically active and could activate the PRL receptor (PRLR). Considering its close phylogenetic homology to PRL and its biological activity in activating the PRLR, we name it as PRL2 and the previously identified PRL as PRL1. In addition, using zebrafish as the animal model, we have further studied the tissue distribution and physiological function of PRL2. The results showed that this novel PRL2 is strikingly different from the classical PRL1 in many aspects.

## Results

### Sequence characterization

In this study, we first searched for the presence of putative *GH* family members in the genomes of elephant shark as well as other distantly related vertebrates and invertebrates. The results showed the absence of *SL*-like genes in elephant shark or lamprey genomes. On the other hand, a *PRL*-like gene (encoding for a protein which we subsequently called PRL2) could be identified in almost all the non-mammalian vertebrate taxa by either bioinformatics or molecular cloning means: Chondrichthyes (elephant shark), Chondrostei (Russian sturgeon, *Acipenser gueldenstaedti*), Teleostei including zebrafish (*Danio rerio*), Nile tilapia (*Oreochromis niloticus*), black seabream (*Acanthopagrus schlegeli*), goldfish (*Carassius auratus*), *Tetraodon* (*Tetraodon nigroviridis*) and medaka (*Oryzias latipes*) (Figures S1,S2,S3,S4,S5,S6,S7–S8). We have also predicted a partial PRL2 sequence in a reptile, the green anole lizard (*Anolis carolinensis*) (Figure S9). Despite our repeated efforts by prediction and cloning, we failed to obtain its full-length sequence. A possible explanation is that this gene has become a pseudogene in lizard. We have also confirmed that an avian PRL, the chicken (*Gallus gallus*) PRLb (NCBI accession no. XP_416043), is in fact a PRL2. In addition we have obtained the full-length cDNA of PRL1 from spotted gar (*Lepisosteus oculatus*) (Figure S10), the coding regions of PRL1 from medaka (Figure S11) and PRL1a from African clawed frog (*Xenopus laevis*) (Figure S12). On the other hand however, PRL2 could not be found in any of the available mammalian genomes indicating that this gene was lost after the divergence that gave rise to the mammalian lineage. Protein sequence comparison shows that PRL2 and PRL1 share a low sequence identity with each other (Table S1A) while PRL2s share a higher identity among themselves (Table S1B).

**Figure 1:**
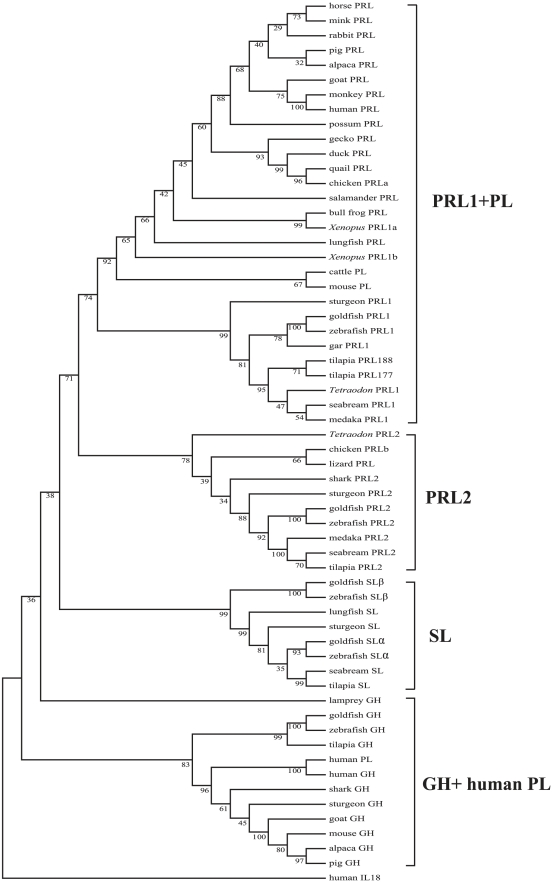
Phylogenetic analysis of PRL, GH, SL and PL sequences. Mega 3.0 was used to construct the tree. The two PRL clades are named as PRL1 and PRL2. All the sequences in the PRL2 clade were identified by us in the present study. The following sequences from the Genbank database were used: human PRL (CAA38264), pig PRL (NP_999091), goat PRL (Q28318), alpaca PRL (ABO21734), rhesus monkey PRL (NP_001040593), possum PRL (O62781), rabbit PRL (NP_001076144), *Xenopus* PRL (NP_001086486, we name this sequence as 1b and the other *Xenopus* PRL as 1a), salamander PRL (AAP93863), gecko PRL (BAD24104), duck PRL (BAD14943), chicken PRLa (NP_9907), horse PRL (P12420), mink PRL (CAA44910), chicken PRLb (XP_416043), quail PRL (BAA83342), bullfrog PRL (CAA34199), marbled lungfish PRL (AAB27569), Russian sturgeon PRL (AAB28396), *Tetraodon* PRL1 (AAR25696), goldfish PRL (AAB47156), black seabream PRL (ABW05297), Nile tilapia PRL188 (CAA00720), human GH (NP_000506), pig GH (NP_999034), goat GH (ACE81811), alpaca GH (ABG67748), Nile tilapia PRL177 (CAA00722), zebrafish PRL1 (NP_852102), goldfish GH (AAC60103), zebrafish GH (NP_001018328), Nile tilapia GH (P13391), Russian sturgeon GH (AAX36064), mouse GH (NP_032143), blue shark GH (P34006), sea lamprey GH (BAC15763), zebrafish SLα (AAR25212), zebrafish SLβ (CAI46892), goldfish SLα (ACB69758), goldfish SLβ (P79697), African lungfish SL (AAC16495), gilthead seabream SL (AAA98734), Mozambique tilapia SL (BAG50585), white sturgeon SL (O93262), cattle PL (NP_851350), human PL (AAA98621), mouse PL (PLAAA39404) and human IL18 (CAG46798). Other PRL1 and all the PRL2 sequences were cloned or predicted in this study with GenBank accession numbers FJ475109-FJ475120.

**Figure 2:**
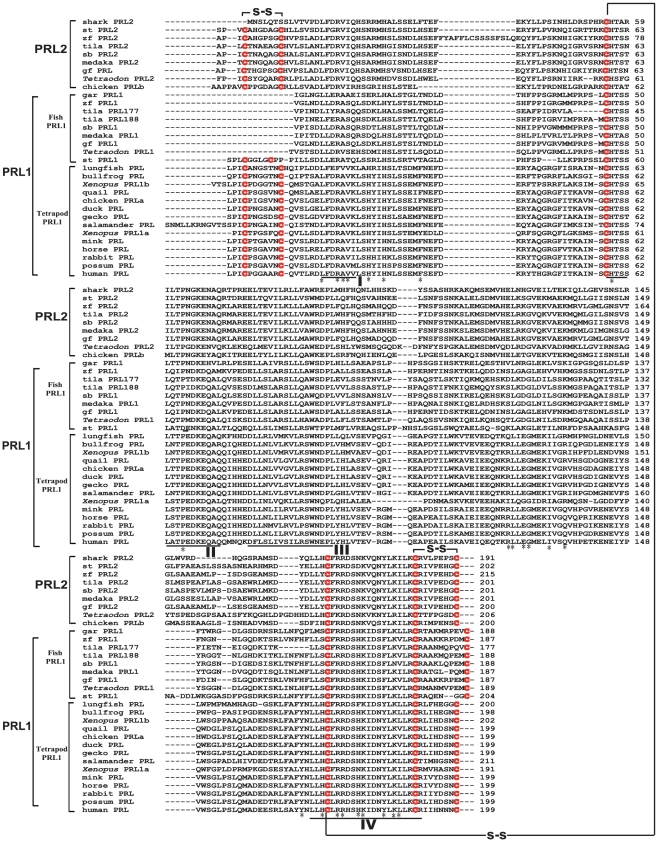
Alignment of PRL1 and PRL2 amino acid sequences. The signal peptides of the sequences were removed. The conserved cysteine residues are highlighted in red. The disulfide linkages are indicated by lines. Four highly conserved segments (I, II, III and IV) are underlined. The conserved amino acids consisting of the two binding sites of human PRL are indicated by *. The accession numbers of the sequences used in this alignment are listed in the legend of Figure 1.

Subsequent to the identification of these PRL2 sequences, phylogenetic analyses were carried out on the different GHs, SLs, PRLs and PLs by three commonly adopted methods of phylogenetic analysis, viz. neighbor-joining (Figure 1), maximum-likelihood (Figure S13) and Bayesian analysis (Figure S14). The results from these three different methods all show that the PRLs are clustered into two separate clades, viz. the PRL1 and PRL2 clades which are distinct from the GH and SL clades. The PRL1 clade contains the classical PRL sequences identified from all the bony fish and tetrapods so far. The PRL2 clade contains the novel PRL sequences obtained from almost all the gnathostomes examined in the present study. Both the *Xenopus* PRL1a and PRL1b belong to the PRL1 clade. The results are also consistent with previous studies that human PL was derived from a duplication of the GH gene [10], while PLs in rodents [11] and ruminants [12] were derived from the PRL gene. A protein sequence alignment of different PRLs was also performed (Figure 2). From this figure, it can be seen that the newly identified PRL2 contains three disulfide linkages with the only exception of the shark PRL2 which contains only two. The teleost PRL1 has lost the first disulfide linkage at the N-terminal.

### Biological characterization of PRL2 in teleost

**Figure 3:**
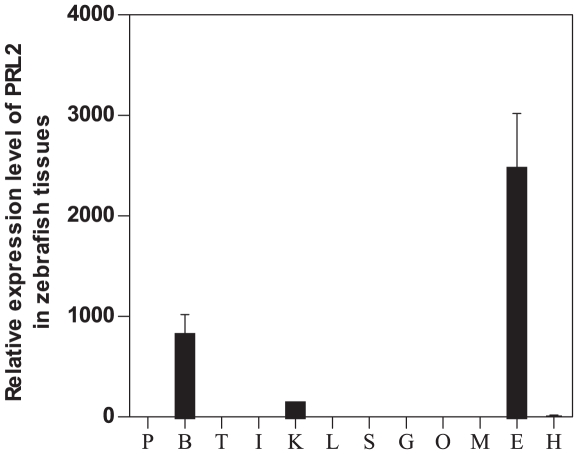
Tissue distribution of PRL2 in zebrafish by real-time PCR. P: pituitary; B: brain; T: testis; I: intestine; K: kidney; L: liver; S: spleen; G: gill; O: ovary; M: muscle; E: eye; H: heart.

**Figure 4:**
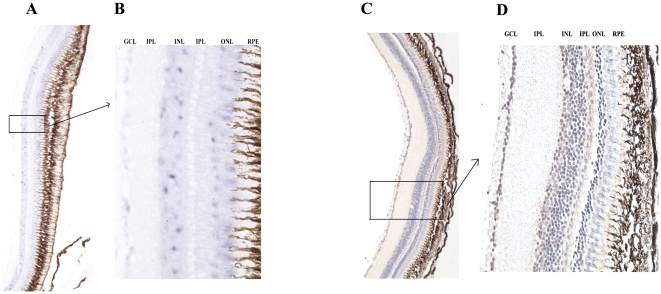
Expression of PRL2 in zebrafish retina. Transverse sections of the zebrafish retina showing expression of zfPRL2 after ISH and IHC. (A and B) ISH signals (gray blue dots) in the GCL, INL and ONL. (C and D) IHC signals (brown dots) in the GCL, INL and ONL. For IHC, nuclei were counterstained with hematoxylin. A and C: ×100; B and D: ×400. IPL: inner plexiform layer; OPL: outer plexiform layer; RPE: retinal pigmented epithelium.

**Figure 5:**
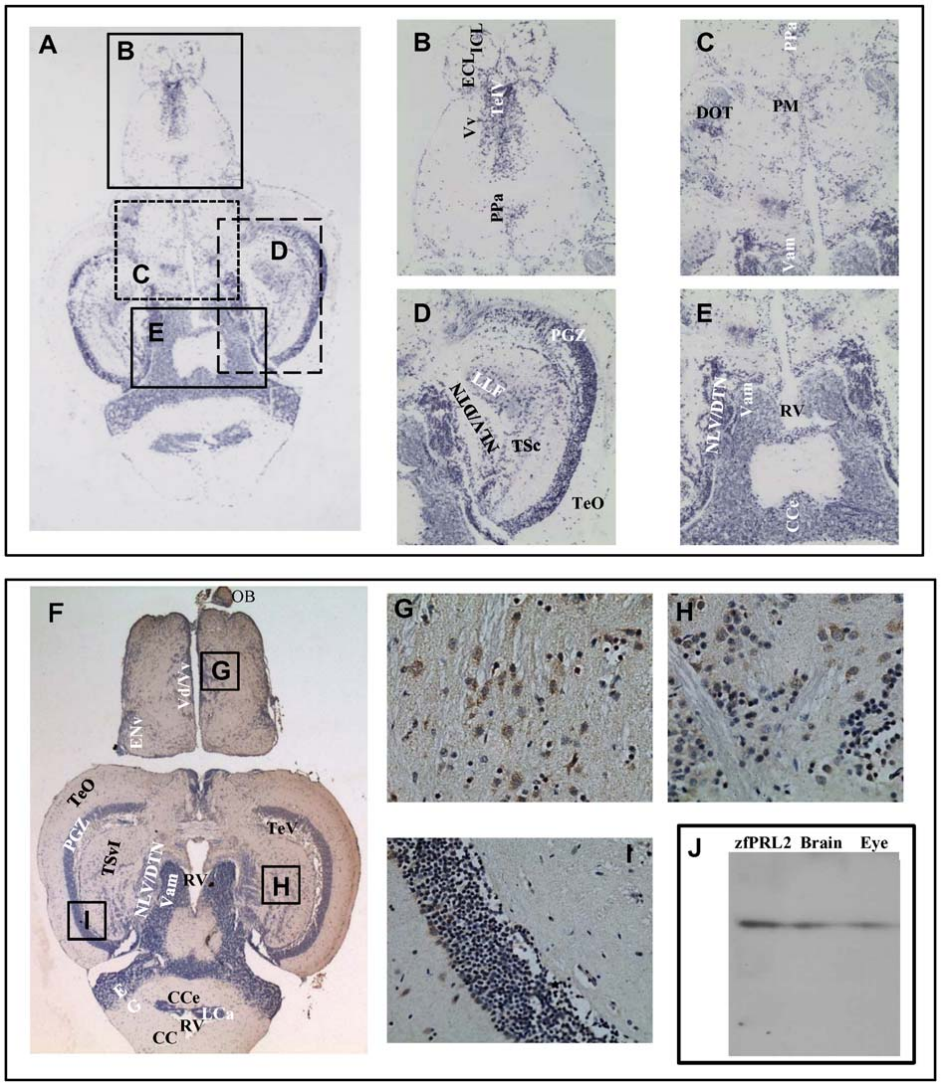
Expression of PRL2 in zebrafish brain. Horizontal sections of the zebrafish brain showing the expression of zfPRL2 after ISH and IHC. (A) An orientation photomicrograph showing the expression of zfPRL2 by ISH in a horizontal section through the zebrafish brain. (B–E) are photographs at higher magnification of the corresponding boxed areas shown in (A). (F) An orientation photomicrograph showing the expression of zfPRL2 by IHC in a horizontal section through the zebrafish brain. Brain regions with positive signals (brown dots) including dorsal nucleus of ventral telencephalic area/ventral nucleus of ventral telencephalic area (Vd/Vv), nucleus lateralis valvulae/dorsal tegmental nucleus (NLV/DTN) and periventricular gray zone of optic tectum (PGZ) are magnified in photomicrographs (G), (H) and (I) respectively. In both the ISH and IHC results, positive signals (dark blue dots) are observed in the following brain regions: corpus cerebella (CCe); dorsomedial optic tract (DOT); external cellular layer of olfactory bulb including mitral cells (ECL); internal cellular layer of olfactory bulb (ICL); lateral longitudinal fascicle (LLF); NLV/DTN; PGZ; magnocellular preoptic nucleus (PM); parvocellular preoptic nucleus, anterior part (PPa); rhombencephalic ventricle (RV); telencephalic ventricles (TeIV); tectum opticum (TeO); central nucleus of torus semicircularis (TSc); medial division of valvula cerebella (Vam); and ventral nucleus of ventral telencephalic area (Vv). Brain regions were identified on a topological atlas of the zebrafish brain [43]. Nuclei were counterstained with hematoxylin. A and F: ×100; B–E: ×200; G–I: ×400. (J) Western blot showing expression of zfPRL2 in zebrafish brain and eye extracts.

Using zebrafish as an animal model, we have analyzed the tissue distribution of this novel PRL2 by real-time PCR. The results show that zfPRL2 is mainly expressed in the eye and brain of zebrafish but not in the pituitary, a pattern which is different from the pituitary members of the GH family (Figure 3). In addition, zfPRL2 is also very weakly expressed in the kidney. The *in situ* hybridization (ISH) and immunohistochemistry (IHC) results showed that zfPRL2 is abundantly expressed in all the three nuclear layers of the retina, i.e. the ganglion cell layer (GCL), the inner nuclear layer (INL) and the outer nuclear layer (ONL), as well as in different parts of the brain (Figures 4 and 5).

**Figure 6:**
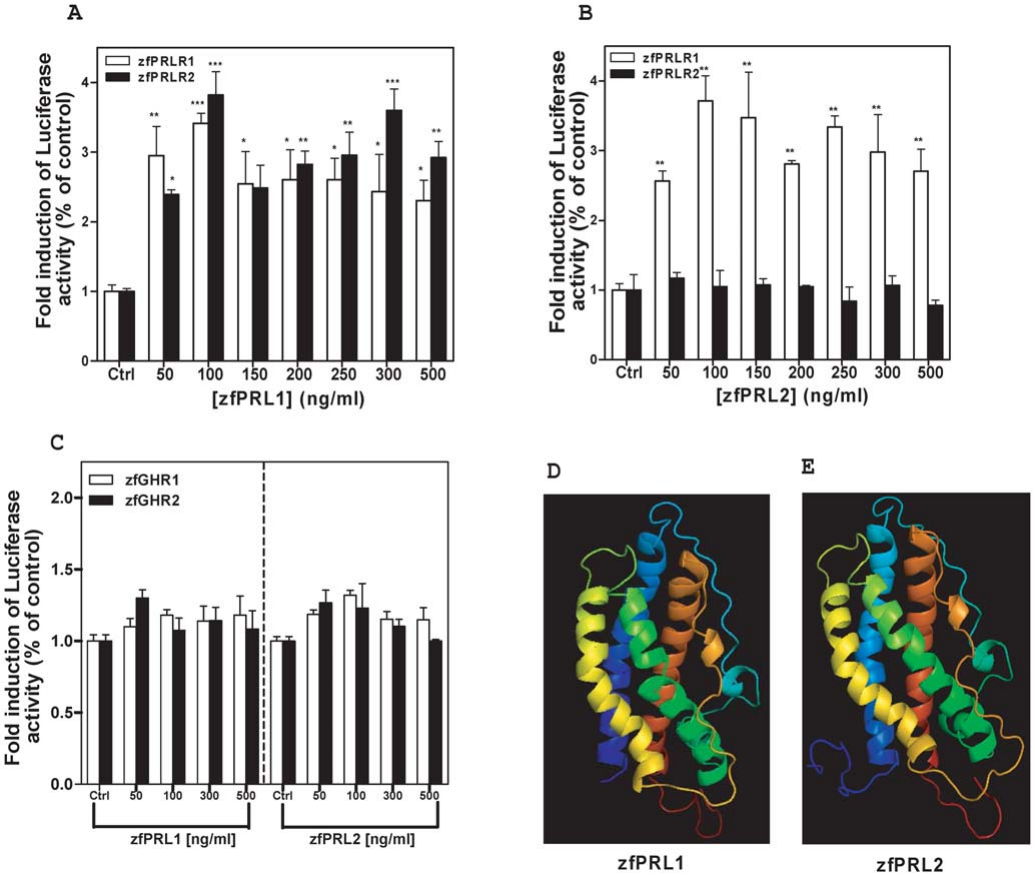
Hormone-receptor interaction studies through receptor transactivation assay. Transactivation of β-casein promoter was conducted in transfected GAKS cells. The cells were co-transfected with a pcDNA3.1 vector containing the entire coding region of either zfPRLR1, zfPRLR2, zfGHR1 or zfGHR2 (A–C) together with a luciferase reporter plasmid driven by the β-casein promoter. The control was the empty pcDNA3.1 vector. The transfected cells were subsequently stimulated by recombinant zfPRL1 or zfPRL2 at different concentrations. Correction for transfection efficiency was performed by measurement of the *Renilla* luciferase activities. Results are mean values±S.E.M. (n = 6; *P<0.05; **P<0.01; ***P<0.001 as compared with the respective controls by one-way ANOVA). The predicted protein structures of zfPRL1 and zfPRL2 (D and E).

Apart from tissue distribution, we have also examined the biological activities of zfPRL2. In this study, recombinant zfPRL1 and zfPRL2 were produced in *E. coli* (Figure S15). The biological activities of these recombinant proteins were examined by a receptor transactivation assay using the Dual Luciferase Reporter Assay system (Promega). Full-length cDNA constructs of the zfPRLR1, zfPRLR2, zfGHR1 and zfGHR2 cloned in an eukaryotic expression vector were individually expressed in cultured GAKS cells together with a reporter gene construct containing a luciferase gene driven by a β-casein promoter. Luciferase activities could be detected in cells expressing zfPRLR1 upon the addition of either recombinant zfPRL1 or zfPRL2, while zfPRLR2 could only be activated by recombinant zfPRL1 (Figure 6A and B). Ligand specificity of receptor interaction was ascertained by parallel studies in which the zfGHR1- and zfGHR2-transfected cells were stimulated with zfPRL1 or zfPRL2. The results showed that zfPRL1 or zfPRL2 were ineffective in activating the zfGHRs (Figure 6C).

### PRL2 in fish retina development

**Figure 7:**
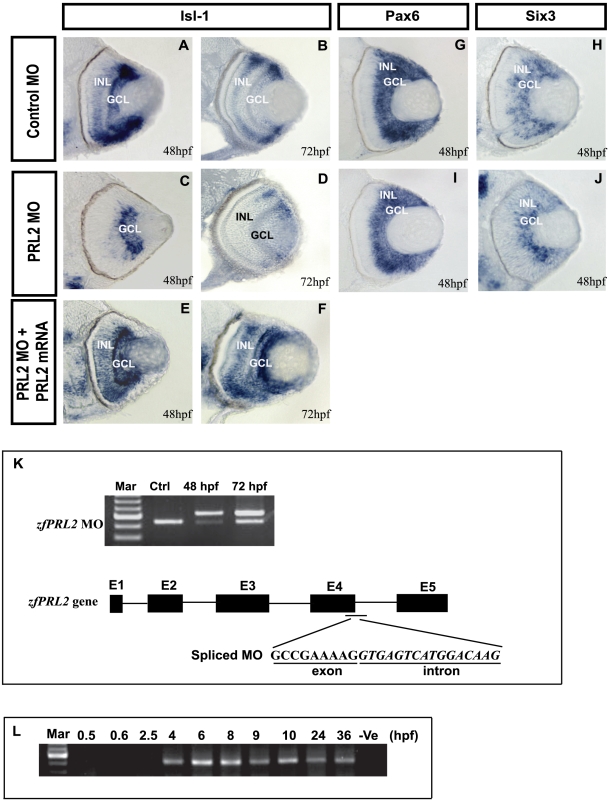
Phenotypic change in the zebrafish eye after knockdown of *zfPRL2*. Expression of *Isl-1* (A–F), *Pax6* (G and I) and *Six3* (H and J) in the zebrafish retina was studied before and after *zfPRL2* MO knockdown. *Isl-1* expression, which is present in the GCL and INL of control MO embryos at 48 hpf (A) and 72 hpf (B), is largely reduced in the INL of *zfPRL2* MO embryos (C and D). This reduction of expression in the INL can be rescued by injection of *zfPRL2* mRNA (E and F). *Pax6* (compare G and I) and *Six3* (compare H and J) expression is however not affected at 48 hpf after *zfPRL2* MO knockdown. After injection of *zfPRL2*MO^s-s^, the transcription of *zfPRL2* was compromised as demonstrated by the aberrant RT-PCR results (K). PRL2 is expressed after 4 hpf in zebrafish embryos (L).

Expression of zfPRL2 could be detected in the early zebrafish embryos (Figure 7L). In view of the unique tissue expression profile of PRL2 in zebrafish, we have also examined the possible involvement of zfPRL2 on eye and brain development in zebrafish. Different gene markers that are important for brain and retina formation and neuronal differentiation were used to check the phenotypic changes after knockdown of *zfPRL2* by morpholino (MO) in zebrafish embryos (Table S2). As shown in Figure 7A and B, *Isl-1* was expressed in the GCL and INL of the wild-type zebrafish eye in 48 and 72 hours post fertilization (hpf) embryos, but was not detected in the INL of the MO morphant (*zfPRL2* ^atg^MO, 85%, *n* = 210; *zfPRL2* ^splice-site(s-s)^MO, 80%, *n* = 235) (Figure 7C and D). The reduction of *Isl-1* expression can be rescued by injection of *zfPRL2* mRNA, indicating that the two *zfPRL2* antisense oligos possess good specificity in the knockdown of *zfPRL2* (Figure 7E and F). The expression of *Pax6* and *Six3* were not altered after the knockdown of *zfPRL2* as compared with the control (Figure 7G–J). Different region-specific brain gene markers were also used to monitor the defects of brain development, but no phenotypic changes were observed (data not shown).

## Discussion

### Evolutionary implication

In this study, we have discovered a *PRL*-like gene in almost all the non-mammalian vertebrates. We name it as PRL2 because of its phylogenetic relationship with the previously identified PRL and its ability to activate PRLR.

In previous studies, sequence analyses showed that all tetrapod PRLs possess three conserved disulfide linkages, while teleost PRLs contain only two. On the basis of this difference, the two-lineage hypothesis has been proposed to account for the evolution of PRL in fish, viz. the tetrapod lineage and the teleost lineage [1], [5], [6]. Our data showed that this hypothesis can only accommodate the classical PRL1 sequences but is not applicable to the novel PRL2 sequences discovered in this study. All the PRL2 sequences possess three conserved disulfide linkages except elephant shark PRL2 which contains only two (Figure 2). But sequence comparison showed that the elephant shark PRL2 actually shares a higher sequence identity with other PRL2s than with PRL1s (Table S1A and B), and this is also consistent with the phylogenetic analyses (Figure 1, SI Figures 13 and 14). However, we could not find PRL1 in elephant shark. The elephant shark PRL2 is the most primitive PRL identified so far. We have in fact searched the genomes of two chordates, viz. the sea squirt (*Ciona intestinalis*) as well as the lancelet (*Branchiostoma floridae*) and a jawless fish, the sea lamprey, but no *PRL*-like genes could be found in these genomes. However, the absence of a *PRL*-like gene in the lamprey genome could be due to the incomplete genome information at the moment.

Interestingly, we have successfully cloned a PRL2 from the most primitive ray-finned fish, the sturgeon. Sequence analysis shows that both this PRL2 and the previously identified sturgeon PRL1 [8] contain three disulfide linkages but share very low sequence identity with each other (34.7%) (Table S1A). Phylogenetic analyses show that this sturgeon PRL2 is orthologous to the shark PRL2 and tetrapod PRL2. Therefore we hypothesize that there exists another PRL (PRL1) in cartilaginous fish as in tetrapods and other fish species. This cartilaginous fish PRL1 may have been lost in elephant shark in a species-specific manner during evolution, or we failed to predict it from the elephant shark genome due to its incomplete genome sequence information, with a 75% coverage till now [4]. Sequences obtained from other cartilaginous fish such as the sharks, rays, skates would be useful to clarify this issue.

To date, gene duplication and loss have been considered to be the primary force of new gene evolution. This phenomenon is evident in a number of sequenced genomes ranging from bacteria to human [13]. The 2R hypothesis is a generally accepted model to explain the evolution of gene families and of vertebrate genomes. In this model, two rounds of genome duplication, often referred to as 1R and 2R, occurred after the divergence of tunicates and lancelets from vertebrates (1R) but before the divergence of cartilaginous fishes and bony vertebrates (2R) [14], [15]. Till now, the most primitive member of the GH family is the GH found in sea lamprey. Other members of the GH family were considered duplication products of this gene [9]. Although the exact time of its occurrence is not completely certain, 2R is believed to have occurred before the divergence of cartilaginous fish and bony fish [4], [16]. According to this genome duplication theory, it is unlikely that the four genes in jawed vertebrates, i.e. GH, PRL1, PRL2 and SL, were duplication products of the single GH in jawless vertebrates. A recent paper reported that 2R occurred before the cyclostomes-gnathostome split [17]. Since none of the GH family members has been found in invertebrates, these genes were very likely derived from 2R concomitantly. The identity and function of the ancestral gene of the GH family is an intriguing question. Answers to this question would await the isolation and study of an orthologue of these four genes in a chordate such as urochordates or cephalochordates where the ancestral gene is not duplicated. An additional round of genome duplication (3R), also known as the fish-specific genome duplication (FSGD), occurred during the evolution of teleost [16], [18]. However, the novel PRL2 in teleost fish was not derived from FSGD because it shows an orthologous relationship with the shark and tetrapod PRL2. We believe that both PRL1 and PRL2 in teleost have undergone FSGD but a copy of the duplicated PRL1 and PRL2 has probably been lost due to its redundant physiological function.

Divergent functions of the duplicated genes might help permanent preservation of the genes. Only if the duplicated genes acquire new functions which are not overlapping with the original gene can the new genes be preserved [19]. Our finding provides another good example to support this gene retention theory. The unique tissue expression pattern of PRL2 and its involvement in the retinogenesis of teleost justify its co-existence with PRL1. The differences between their physiological functions necessitate the need for keeping both genes in non-mammalian vertebrates. However, the reason for the loss of the PRL2 gene in mammals during evolution is not known.

The existence of more than one form of PRL has been reported previously in some vertebrate taxa, including teleost [20]–[25], amphibian [26], rodents [27] and primates [28]. All these multiple forms of PRLs are very similar to each other and they all belong to the PRL1 clade. They are likely the products of single or multiple gene duplication in each specific species and are entirely different from the novel PRL2 reported in the present study. Besides fish, we also found a partial PRL2 sequence in green lizard and confirmed that the chicken PRLb belongs to the PRL2 (Figure 1).

### Receptor transactivation assay

In the reporter transactivation assays, only zfPRL1 can trigger the downstream post-receptor event via interacting with both zfPRLR1 and zfPRLR2. However, zfPRL2 is only active towards zfPRLR1 (Figure 6B). In addition, zfPRL1 and zfPRL2 are inactive towards zfGHR1 and zfGHR2, indicating their specificity of action. The biological activities of PRL are mediated through binding to PRLR in a one ligand-two receptors proportionality in a manner determined by the tertiary structure of the ligand and receptors [7]. PRL contains two receptor binding sites which sequentially interact with two PRLR molecules to form a ternary complex. Comparing the two binding sites of zfPRL1 and zfPRL2 with human PRL [29] (Figure 2), most of the amino acids are conserved. Besides, the predicted protein structures of both zfPRL1 and zfPRL2 are similar to each other (Figure 6D and E). Whether the subtle difference in protein structure between zfPRL1 and zfPRL2 would result in differential receptor binding affinity towards the two receptors or in their preference for signaling pathway usage awaits further experimentation.

### Functional significance in eye development

An early and fundamental step for retina development is the generation of distinct retinal cell types in appropriate numbers. These distinct cell types are first generated from the retinal progenitor cells in the neuroblastic layer and then orderly establish the three major nuclear layers of the retina: GCL, INL and ONL [30]. Till now, many different factors are known to be involved in retinogenesis. *Pax6* and *Six3* are eye-determination genes involved in the early processes of induction and regional specification [31]. In our study, after the knockdown of *zfPRL2*, the expression levels of both *Pax6* and *Six3* were not altered, indicating that early eye formation was not compromised. *Isl-1* has an indispensable role in retinal neuron differentiation [32]. The expression of *Isl-1* was largely reduced suggesting that *zfPRL2* may play an important role in neuron differentiation in the INL. To our knowledge, this is the first report on the identification of a factor that specifically regulates neuron differentiation in the INL of zebrafish. Compared with the observed effects of *zfPRL1* knockdown on embryogenesis, viz. defects in gas bladder inflation, reduced head and eye size, shorter body length and fewer melanophores [33], *zfPRL2* possesses specific roles in retina development.

**Figure 8:**
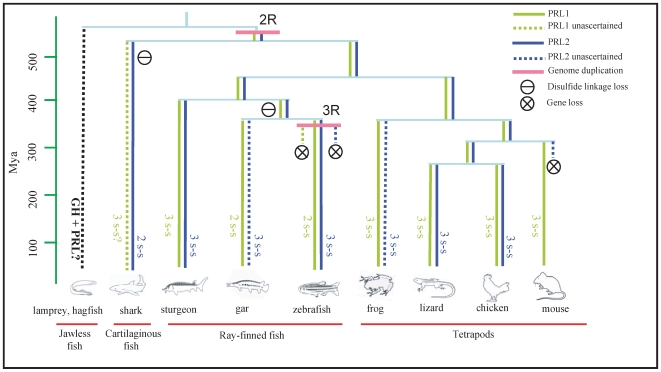
A proposed evolutionary scheme for PRL1 and PRL2. Labeling of genes is shown in the key. Unascertained places are indicated by question marks and dotted lines. Time for divergence in millions of years (Mya) ago was taken from [44], [45]. 2R and 3R stand for the second and third round of genome duplication respectively.

In summary, a new PRL was discovered in non-mammalian jawed vertebrates. The most primitive PRL identified so far is the cartilaginous PRL2 found in the present study. In this study, we have also proposed a new hypothesis on the evolution of PRLs in vertebrates (Figure 8). These results will provide new avenues of research on the neuroendocrine control of eye development in vertebrates.

## Materials and Methods

### Animals

All animals were purchased from local markets. Elephant shark eye and brain cDNAs were gifts from Dr. Byrappa Venkatesh at the Institute of Molecular and Cell Biology in Singapore. Adult wild-type zebrafish were maintained at 28°C on a 14 h∶10 h (light∶dark) cycle, and fed twice daily. Embryos were generated from natural crosses. Fertilized eggs were raised in embryo medium at 28.5°C. All experiments were conducted in accordance with guidelines established by the University Committee on the use and care of laboratory animals at The Chinese University of Hong Kong.

### Data mining, PCR cloning and phylogenetic analysis

By searching the genome databases of zebrafish, medaka, *Tetraodon nigroviridis*, (Ensembl genome browser: http://www.ensembl.org/index.html and NCBI database: http://www.ncbi.nlm.nih.gov/), elephant shark (http://esharkgenome.imcb.a-star.edu.sg) and green anole lizard (http://genome.ucsc.edu/cgi-bin/hgGateway?clade=other&org=Lizard), putative PRL1 and PRL2 sequences were identified and then confirmed by RT-PCR. PRL2s in other animals were cloned by RT-PCR and RACE techniques. The primers used are listed in Table S2. Phylogenetic trees were constructed independently by three different methods, viz. the neighbor-joining method using Mega 4.0 [34], and the maximum likelihood method using Phylip 3.68 [35], and the Bayesian analysis using MrBayes framework 3.1.2 [36].

### Tissue distribution, ISH and IHC

Tissue distribution of zfPRL2 was carried out by real-time PCR. The methods were carried out as described by Huang *et al.* [37]. For ISH and IHC, the eye and brain of adult zebrafish were dissected out and fixed in 4% paraformaldehyde (PFA) in 0.1 M PBS at 4°C overnight. After fixation, tissues were embedded in paraffin wax. Sections were cut at 5 µm thickness. ISH and IHC were carried out as previously described [38].

### Expression of recombinant zfPRL1 and zfPRL2 in *E. coli* and production of zfPRL2 polyclonal antibody

The signal peptides of zfPRL1 and zfPRL2 were predicted by SignalP 3.0 (http://www.cbs.dtu.dk/services/SignalP/.Primers). The mature peptide coding regions were inserted into a prokaryotic SUMO vector (a gift from Dr. Shannon W. N. Au at The Chinese University of Hong Kong) by taking advantage of the *Bam*HI/*Eco*RI sites of the SUMO vector. After protein induction and purification [39], the polypeptide was recovered through removing the SUMO tag on a nickel column by virtue of its hexa-His motif after a SUMO-specific protease SENP1 (sentrin-specific protease 1) digestion from the fusion protein. Polyclonal antibodies against the recombinant zfPRL2 were prepared in New Zealand White rabbits as previously described [40]. The specificity of the antibody was checked by Western blot. The protein structures of zfPRL1 and zfPRL2 were predicted and viewed by Phyre (http://www.sbg.bio.ic.ac.uk/phyre/) and PyMOL (http://pymol.sourceforge.net/).

### Functional studies of recombinant zfPRLs

The entire coding regions of zfPRLR1 (NCBI accession no. NM_001128677.1), zfPRLR2 (NCBI accession no. NM_001113500), zfGHR1 (NCBI accession no. EU649774) and zfGHR2 cDNA (NCBI accession no. NM_001111081) were inserted into pcDNA3.1 (Invitrogen). Fifty nanogram of each construct together with 500 ng of a luciferase reporter plasmid containing the rat β-casein promoter and 20 ng of the pRL/CMV vector as a control for transfection efficiency were transiently transfected into goldfish scale fibroblast GAKS cells (ATCC). The hormone treatment and the subsequent measurement of luciferase activities were carried out as previously described [37].

### Morpholino and capped mRNA injections

Two antisense MO oligonucleotides (zfPRL2^atg^MO and zfPRL2^s-s^MO) for *zfPRL2* and one control MO were obtained from GeneTools (GeneTools, Oregon). Capped mRNA was transcribed with SP6 RNA polymerase using the mMessage mMachine Kit (Ambion). After injection, embryos were maintained in fish medium with PTU and fixed with 4% PFA/PBS at 48 hpf and 72 hpf. Embryos were embedded in OCT medium for cryosection at 15 µm [41]. The following gene markers were used in the whole mount ISH: *Pax6*, *Six3* and *Isl-1* for retina [31], [32]; *emx1* for dorsal anterior forebrain; *dlx2* for forebrain; *otx2* for midbrain; *pax2.1* for mid-hindbrain boundary and *krox20* for hindbrain [42]. The average frequency (%) was deduced from 3 independent microinjection experiments and the total number (n) is >200.

### Data analysis

Data are shown as mean values±SEM of duplicated measurements from at least three independent experiments. All data were analyzed by one-way ANOVA followed by a Turkey's test on the PRISM software (Version 3.0; GraphPad, San Diego, CA).

### Data deposition

The sequences reported in this paper have been deposited in the GenBank database [accession nos: FJ475109-FJ475120].

## Supporting Information

Table S1 Amino acid identity of PRL1s and PRL2s in different species.
(0.05 MB DOC)

Table S2 Primer sequences used in the present study.
(0.10 MB DOC)

Figure S1 The cDNA and amino acid sequences of shark PRL. The cDNA sequence was cloned from elephant shark eye. The numbers on the right are the positions of the nucleotide sequence. Stop codon is represented by an asterisk. The shark PRL cDNA encompasses a 117-bp 5′ UTR, a 636-bp ORF encoding a 211-aa protein. The prepro-shark PRL has a putative 20-aa signal peptide (underlined) and 4 conserved cysteine residues (enclosed by squares).
(0.24 MB EPS)

Figure S2 The cDNA and amino acid sequences of sturgeon PRL2. The cDNA sequence was cloned from sturgeon eye. The numbers on the right are the positions of the nucleotide sequence. Stop codon is represented by an asterisk. The sturgeon PRL2 encompasses a 30-bp 5′ UTR, a 675-bp ORF encoding a 224-aa protein and a 301-bp 3′ UTR. The prepro-sturgeon PRL2 has a putative 22-aa protein signal peptide (underlined) and 6 conserved cysteine residues (enclosed by squares).
(0.36 MB EPS)

Figure S3 The cDNA and amino acid sequences of zebrafish PRL2. The cDNA sequence was cloned from zebrafish eye. The numbers on the right are the positions of the nucleotide sequence. Stop codon is represented by an asterisk. The zebrafish PRL2 cDNA encompasses a 30-bp 5′ UTR, a 678-bp ORF encoding a 225-aa protein, and a 259-bp 3′ UTR. The prepro-zebrafish PRL2 has a putative 26-aa signal peptide (underlined) and 6 conserved cysteine residues (enclosed by squares).
(0.36 MB EPS)

Figure S4 The cDNA and amino acid sequences of Nile tilapia PRL2. The cDNA sequence was cloned from Nile tilapia eye. The numbers on the right are the positions of the nucleotide sequence. Stop codon is represented by an asterisk. The Nile tilapia PRL2 cDNA encompasses a 48-bp 5′ UTR, 690-bp ORF encoding a 229-aa protein, and a 254-bp 3′ UTR. The prepro-tilapia PRL2 has a putative 29-aa signal peptide (underlined) and 6 conserved cysteine residues (enclosed by squares).
(0.36 MB EPS)

Figure S5 The cDNA and amino acid sequences of black seabream PRL2. The cDNA sequence was cloned from black seabream eye. The numbers on the right are the positions of the nucleotide sequence. Stop codon is represented by an asterisk. The seabream PRL2 cDNA encompasses a 96-bp 5′ UTR, 690-bp ORF encoding a 229-aa protein, and a 350-bp 3′ UTR. The prepro-seabream PRL2 has a putative 29-aa signal peptide (underlined) and 6 conserved cysteine residues (enclosed by squares).
(0.36 MB EPS)

Figure S6 The cDNA and amino acid sequences of goldfish PRL2. The cDNA sequence was cloned from goldfish eye. The numbers on the right are the positions of the nucleotide sequence. Stop codon is represented by an asterisk. The goldfish PRL2 cDNA encompasses a 43-bp 5′ UTR, a 678-bp ORF encoding a 225-aa protein, and a 195-bp 3′ UTR. The prepro-goldfish PRL2 has a putative 26-aa signal peptide (underlined) and 6 conserved cysteine residues (enclosed by squares).
(0.36 MB EPS)

Figure S7 The cDNA sequence assembled by EST and amino acid sequence of Tetraodon PRL2. The numbers on the right are the positions of the nucleotide sequence. Stop codon is represented by an asterisk. The Tetraodon PRL2 cDNA encompasses a 672-bp ORF encoding a 223-aa protein, and a 68-bp 3′ UTR. The prepro-Tetraodon PRL2 has a putative 18-aa signal peptide (underlined) and 6 conserved cysteine residues (enclosed by squares).
(0.46 MB EPS)

Figure S8 The cDNA and amino acid sequences of medaka PRL2. The cDNA sequence was predicted from the medaka's genome. The numbers on the right are the positions of the nucleotide sequence. Stop codon is represented by an asterisk. The medaka PRL2 cDNA encompasses a 681-bp ORF encoding a 226-aa protein. The prepro-medaka PRL2 has a putative 26-aa signal peptide (underlined) and 6 conserved cysteins residues (enclosed by squares).
(0.36 MB EPS)

Figure S9 A partial fragment of the green anole lizard PRL2 was predicted from its genome. This fragment encompasses 198-bp encoding a 66-aa protein sequence. Homology analysis demonstrated that this fragment is highly homologous to the corresponding part of other PRL2s. Phylogenetic analysis also showed that it belongs to the PRL2 clade.
(0.35 MB EPS)

Figure S10 The cDNA and amino acid sequences of spotted gar PRL1. The cDNA sequence was cloned from the spotted gar pituitary. The numbers on the right are the positions of the nucleotide sequence. Stop codon is represented by an asterisk. The gar PRL1 encompasses a 298-bp 5′ UTR, a 648-bp ORF encoding a 215-aa protein, and a 1122-bp 3′ UTR. The prepro-gar PRL1 has a putative 27-aa signal peptide (underlined) and 4 conserved cysteine residues (enclosed by squares).
(0.37 MB EPS)

Figure S11 The cDNA and amino acid sequences of medaka PRL1. The cDNA sequence was predicted from the medaka's genome. The numbers on the right are the positions of the nucleotide sequence. Stop codon is represented by an asterisk. The medaka PRL1 ORF encompasses 639 bp encoding a 212-aa protein. The prepro-medaka PRL1 has a putative 25-aa signal peptide (underlined) and 4 conserved cysteine residues (enclosed by squares).
(0.36 MB EPS)

Figure S12 The cDNA and amino acid sequences of Xenopus PRL1a. The cDNA sequence was predicted from the Xenopus's genome. The numbers on the right are the positions of the nucleotide sequence. Stop codon is represented by an asterisk. The Xenopus PRL1 ORF encompasses 636 bp encoding a 211-aa protein. The prepro-Xenopus PRL1 has a putative 20-aa signal peptide (underlined) and 6 conserved cysteine residues (enclosed by squares).
(0.45 MB EPS)

Figure S13 Phylogenetic analysis of PRLs, GHs, SLs, and PLs. Maximum likelihood method was used to calculate the tree. Treeview was used to view the tree. Sequences are the same as those used in Figure 1.
(1.25 MB EPS)

Figure S14 Phylogenetic analysis of PRLs, GHs, SLs, and PLs. Bayesian method was used to calculate the tree. Treeview was used to view the tree. Sequences are the same as those used in Figure 1.
(1.22 MB EPS)

Figure S15 SDS-PAGE gel pictures showing the production of recombinant zebrafish PRL1 (A and B) and PRL2 (C and D). (A) Lane 1: BL21 cell lysate; lane 2: column flow-through; lane 3: column wash with 50 mM PBS; lanes 4–7: column wash with 25 mM, 50 mM, 100 mM and 500 mM imidazole respectively. The arrow indicates the collected zfPRL1-SUMO fusion protein. (B) Lane 1: enzyme digestion mixture of zfPRL1-SUMO fusion protein by SENP1; lane 2: column wash with PBS; lanes 3–5: column wash with 50 mM, 100 mM and 500 mM imidazole respectively. The arrow indicates the collected zfPRL1 protein. (C) Lane 1: BL21 cell lysate; lane 2 column flow-through; lane 3: column wash with 50 mM PBS; lanes 4–7: column wash with 25 mM, 50 mM, 100 mM and 500 mM imidazole respectively. The arrow indicates the collected zfPRL2-SUMO fusion protein. (D) Lane 1: enzyme digestion mixture of zfPRL2-SUMO fusion protein by SENP1; lane 2: column flow-through; lanes 3-5: column wash with 20 mM, 50 mM, 100 mM imidazole respectively. The arrow indicates the collected zfPRL2 protein. M stands for protein markers used in the SDS-PAGE.
(6.74 MB EPS)
