# Supplementary material for: Discovery of a Novel Prolactin in Non-Mammalian Vertebrates: Evolutionary Perspectives and Its Involvement in Teleost Retina Development
Source: PLoS One. 2009 Jul 8;4(7):e6163. doi: 10.1371/journal.pone.0006163 (PMC2702173; doi:10.1371/journal.pone.0006163)
Supplement: Table S1 — Amino acid identity of PRL1s and PRL2s in different species. (0.05 MB DOC) [file pone.0006163.s001.doc]

**Table S1**A

|  | PRL2 | | | | | | | | |
| --- | --- | --- | --- | --- | --- | --- | --- | --- | --- |
| PRL1 | shark | sturgeon | *Tetraodon* | tilapia | goldfish | medaka | seabream | zebrafish | chicken b |
| rabbit | 32.4 | 36.0 | 32.8 | 31.6 | 35.5 | 33.2 | 33.2 | 35.5 | 34.2 |
| sturgeon | 27.7 | 34.7 | 28.8 | 31.2 | 31.6 | 33.3 | 30.7 | 32.6 | 28.6 |
| *Tetraodon* | 29.8 | 32.6 | 30.2 | 31.5 | 32.0 | 30.9 | 30.9 | 33.1 | 32.2 |
| tilapia | 28.1 | 30.9 | 28.5 | 30.9 | 31.5 | 30.9 | 30.9 | 32.6 | 28.2 |
| goldfish | 28.7 | 32.6 | 29.1 | 32.0 | 32.0 | 32.0 | 33.1 | 33.7 | 31.1 |
| medaka | 31.6 | 32.8 | 29.2 | 31.7 | 31.1 | 32.2 | 31.7 | 32.2 | 31.2 |
| seabream | 29.8 | 30.9 | 27.9 | 31.5 | 31.5 | 30.9 | 30.9 | 32.6 | 29.4 |
| zebrafish | 29.2 | 31.5 | 29.1 | 32.6 | 32.0 | 32.6 | 33.1 | 33.7 | 31.1 |
| chicken a | 30.3 | 33.5 | 31.8 | 30.5 | 33.5 | 32.1 | 31.6 | 33.5 | 30.6 |

Amino acid identity of PRL1s and PRL2s in different species. The Jotun Hein method of the DNASTAR software was employed to compare the polypeptide sequences. Data are expressed as percentage aa identity.

**Table S1B**

Amino acid identity of PRL2s in different species. The Jotun Hein method of the DNASTAR software was employed to compare the polypeptide sequences. Data are expressed as percentage aa identity.

|  | PRL2 | | | | | | | | |
| --- | --- | --- | --- | --- | --- | --- | --- | --- | --- |
| PRL2 | shark | sturgeon | *Tetraodon* | tilapia | goldfish | medaka | seabream | zebrafish | chicken b |
| shark | 100 |  |  |  |  |  |  |  |  |
| sturgeon | 59.9 | 100 |  |  |  |  |  |  |  |
| *Tetraodon* | 45.5 | 51.5 | 100 |  |  |  |  |  |  |
| tilapia | 56.8 | 68.5 | 51.3 | 100 |  |  |  |  |  |
| goldfish | 55.7 | 68.7 | 56.1 | 75.0 | 100 |  |  |  |  |
| medaka | 55.7 | 70.5 | 52.3 | 86.5 | 75.0 | 100 |  |  |  |
| seabream | 56.8 | 70.0 | 54.8 | 87.1 | 74.5 | 85.1 | 100 |  |  |
| zebrafish | 56.2 | 69.2 | 56.1 | 73.5 | 95.5 | 72.5 | 75.0 | 100 |  |
| chicken b | 55.9 | 58.9 | 43.8 | 54.6 | 53.8 | 55.1 | 57.7 | 55.3 | 100 |
