# Supplementary material for: Discovery of a Novel Prolactin in Non-Mammalian Vertebrates: Evolutionary Perspectives and Its Involvement in Teleost Retina Development
Source: PLoS One. 2009 Jul 8;4(7):e6163. doi: 10.1371/journal.pone.0006163 (PMC2702173; doi:10.1371/journal.pone.0006163)
Supplement: Table S2 — Primer sequences used in the present study. (0.10 MB DOC) [file pone.0006163.s002.doc]

**Table S2**

Primer sequences used in the present study. The abbreviations sb, zf, nt, gf, st stand for black seabream, zebrafish, Nile tilapia, goldfish and sturgeon.

| Purpose | Primer name | Sequence (5’ to 3’) |
| --- | --- | --- |
| ***Seabream, zebrafish, tilapia and goldfish PRL2 sequences*** | | |
| Partial | PRL2-F1 | GAGCWKTACTTCMTGCCCAGYAAGAA |
|  | PRL2-F2 | ACCTCMAGMATCCTCACHCCAAATGG |
|  | PRL2-F3 | GAGGTGATCCTVAAGCTGYTGGTGG |
|  | PRL2-R1 | ACCTTTSCKNAGCTCRTGCACCATGT |
|  | PRL2-R2 | CBCKGCGAAAGCAGTASAGGAG |
|  | PRL2-R3 | CACTTSAGGATCTTCAGGTAGTT |
| sbPRL2 5’RACE | sbPRL2-R2 | CCGTGCATTCTGGCTGAGTGTTG |
|  | sbPRL2-R3 | CGCCACTCAGCACTGTCAGATG |
| sbPRL2 3’RACE | sbPRL2-F2 | GTGGGTGGTGTTGGTCTAT |
|  | sbPRL2-F3 | TGACGGAGGTGATTCTGA |
| sbPRL2 full-length | sbPRL2-UTR-F | TTCTACTCTGAGACAGGAGCTC |
|  | sbPRL2-UTR-R | TCAGACACGAAATGGAGAATGGGTC |
| zfPRL2 5’RACE | zfPRL2-R2 | CTGTTCGACAGGGTCATCCAG |
|  | zfPRL2-R3 | CCAGCAGGACTTCAACAG |
| zfPRL2 3’RACE | zfPRL2-F2 | TCAGGTTTATTTACTGGGATT |
|  | zfPRL2-F3 | TCACTGAGTTCCCAAGCATC |
| zfPRL2 full-length | zfPRL2-UTR-F | CACAACCTCTCCTTCTCCTCTCC |
|  | zfPRL2-UTR-R | CTTGTGGACCAGCGTGTGTCCAT |
| ntPRL2 5’RACE | ntPRL2-R2 | GTAGTTGAGAAGATGCAGATG |
|  | ntPRL2-R3 | GTCAGCAGCCTCATGTCTCC |
| ntPRL2 3’RACE | ntPRL2-F2 | TGGTGGAAATGCCACAGAGG |
|  | ntPRL2-F3 | AATCACCTCCGTCAGCTCCTC |
| ntPRL2 full-length | ntPRL2-UTR-F | TGCTCTAAGACAGGATCTCCT |
|  | ntPRL2-UTR-R | ACCCTGTCTGTGGTCATGTAACAT |
| gfPRL2 5’ RACE | gfPRL2-R3 | GTTGAAGTCCTGCTGGTCAGC |
|  | gfPRL2-R4 | CTCTGGTGCAGCTGGAACAGG |
| gfPRL2 3’ RACE | gfPRL2-F3 | GTGAAGAGTTAACGGAGGTG |
|  | gfPRL2-F4 | CAGAGCATGGCTGACCAGCAGG |
| gfPRL2 full-length | gfPRL2-UTR-F | CTCTCAGAATGTCTAGGAGC |
|  | gfPRL2-UTR-R | GTGTCCCATTTGCTGTCCAG |
| zfPRL2 tissue distribution | zfReal-F1 | GCGGCGAAAGCAGTAGAG |
|  | zfReal-R1 | GCGGCGAAAGCAGTAGAG |
| zfGAPDH | zfGAPDH-F1 | GTGTAGGCGTGGACTGTGGT |
|  | zfGAPDH-R1 | TGGGAGTCAACCAGGACAAATA |
| ***Recombinant proteins*** | | |
| zfGH | zfGH-SUMO-F | ACCGGTGGATCCGAAAACCAGCGGCTCTTCAA |
|  | zfGH-SUMO-R | GGTACCCTACAGGGTACAGTTGGAATCCAG |
| zfSLβ | zfSLβ-SUMO-F | ACCGGTGGATCTCCAGTGGAGTGTCCAGACCA |
|  | zfSLβ-SUMO-R | GGTACCCTAGAAGAGGGAGCAGTTTTCCCTGT |
| zfPRL1 | zfPRL1-SUMO-F | ACCGGTGGAGTGGGTCTGAATGATTTG |
|  | zfPRL1-SUMO-R | GGTACCCTAGCACATGTCAGGCCTC |
| zfPRL2 | zfPRL2-SUMO-F | ACCGGTGGAGCACCTATCTGTGCTCAC |
|  | zfPRL2-SUMO-R | GGTACCCTAACAGTCATGTTCGGGT |
| ***Gar PRL1 sequence*** | | |
| Partial | garPRL1-F1 | CGCCCCTCTMTSTGYCACACCTCCTC |
|  | garPRL1-R1 | CTGTCAATTTTGTGGGAGTCCC |
| gar PRL1 5’ RACE | garPRL1-R2 | GCTCCATCCACAGCAAGACC |
|  | garPRL1-R3 | CTGGAGAAGCTGGTCAGCAAG |
|  | garPRL1-R4 | CTTCCAGTTCCTGATGTCCTG |
| gar PRL1 3’RACE | garPRL1-F2 | TGCCTCAGCAGACAGCAGGTG |
|  | garPRL1-F3 | GTCACTCCAGGACAGCAGCAG |
|  | garPRL1-F4 | CTTGTCGTTGGGTGTGGTCAG |
| ***Shark PRL sequence*** | | |
| shark PRL 5’ RACE | sharkPRL-R1 | CTCTAACCCACAAGCCTCTCAG |
|  | sharkPRL-R2 | GTTCAGCTCATGCACCATCTC |
|  | sharkPRL-R3 | GATCACCTCCGTCAACTCCTC |
|  | sharkPRL-R4 | TCAGCAGCTGGGCTCTGGCAGGAC |
| ***Sturgeon PRL2 sequence*** | | |
| Partial | stPRL2-F1 | ACYCCRAAYGGNAARGASAAYGC |
|  | stPRL2-F2 | GARGAGYTRACNGAGGTGAT |
|  | stPRL2-F3 | ATGRGNRANATGGYSCAYGAGCT |
|  | stPRL2-R1 | CAARRTSCARAAYTAYCT |
|  | stPRL2-R2 | AAYTAYCTRAARATCYTSAAGTG |
| stPRL2 3’RACE | stPRL2-RACE-F1 | TGGACTGTTTCCTGCTGAAGC |
|  | stPRL2-RACE-F2 | GTCTGCCAGCAATGAAGCAAGG |
|  | stPRL2-RACE-F3 | GAAGCAAGGCATATGAGAGAC |
| stPRL2 5’RACE | stPRL2-RACE-R1 | CAATGAAGCAATTCATAGTCTCTC |
|  | stPRL2-RACE-R2 | CTTGCTTCATTGCTGGCAGAC |
|  | stPRL2-RACE-R3 | CTTGCTTCAGCAGGAAACAGTC |
| stPRL2 full-length | stPRL2-5’UTR-F | AGCAAGATTTGAAGTAAGTCCAG |
|  | stPRL2-3’UTR-R | ACGAACCCTTAAACCGCCAGCGG |
| ***Knockdown experiments*** | | |
| Start codon MO | zfPRL2^atg^MO | CCACTTGCTTCAGGCTCCTAGACAT |
| Splice junction MO | zfPRL2^s-s^MO | CTTGTCCATGACTCACCTTTTCGGC |
| Control MO | zfPRL2 ^ctrl^MO | CTTCTCGATGAGTCACGTTTTCCGC |
| ***Others*** | | |
| Anchor primers for RACE | AP | GGCCACGCGTCGACTAGTAC(T)16 |
|  | AAP | GGCCACGCGTCGACTAGTACGGGGGGGGGG |
|  | AUAP | GGCCACGCGTCGACTAGTAC |
| Vector primers | T7 | TAATACGACTCACTATAGGG |
|  | SP6 | ATTTAGGTGACACTATAGAA |
| SMART RACE primers | UPM | CTAATACGACTCACTATAGGGC |
|  | NUP | AAGCAGTGGTATCAACGCAGAGT |
| β-actin | Actin-F | ACCCAGATCATGTTCGAGACC |
|  | Actin-R | ATGAGGTAGTCTGTGAGGTCG |
